# A Web Application for Biomedical Text Mining of Scientific Literature Associated with Coronavirus-Related Syndromes: Coronavirus Finder

**DOI:** 10.3390/diagnostics12040887

**Published:** 2022-04-02

**Authors:** Dagoberto Armenta-Medina, Aniel Jessica Leticia Brambila-Tapia, Sabino Miranda-Jiménez, Edel Rafael Rodea-Montero

**Affiliations:** 1 Consejo Nacional de Ciencia y Tecnología (CONACyT), Ciudad de México 03940, Mexico; sabino.miranda@infotec.mx; 2 Centro de Investigación e Innovación en Tecnologías de la Información y Comunicación (INFOTEC), Aguascalientes 20326, Mexico; 3 Centro Universitario de Ciencias de la Salud (CUCS), Departamento de Psicología Básica, Universidad de Guadalajara, Guadalajara 44340, Mexico; aniel.brambila@academicos.udg.mx; 4 Hospital Regional de Alta Especialidad del Bajío, León 37660, Mexico; edel.rodea@hraeb.gob.mx

**Keywords:** coronavirus, natural language processing, latent semantic analysis, SARS, MERS

## Abstract

In this study, a web application was developed that comprises scientific literature associated with the *Coronaviridae* family, specifically for those viruses that are members of the Genus Betacoronavirus, responsible for emerging diseases with a great impact on human health: Middle East Respiratory Syndrome-Related Coronavirus (MERS-CoV) and Severe Acute Respiratory Syndrome-Related Coronavirus (SARS-CoV, SARS-CoV-2). The information compiled on this webserver aims to understand the basics of these viruses’ infection, and the nature of their pathogenesis, enabling the identification of molecular and cellular components that may function as potential targets on the design and development of successful treatments for the diseases associated with the *Coronaviridae* family. Some of the web application’s primary functions are searching for keywords within the scientific literature, natural language processing for the extraction of genes and words, the generation and visualization of gene networks associated with viral diseases derived from the analysis of latent semantic space, and cosine similarity measures. Interestingly, our gene association analysis reveals drug targets in understudies, and new targets suggested in the scientific literature to treat coronavirus.

## 1. Introduction

Coronaviruses have been associated with human respiratory infections since the mid-20th century [1,2]. Coronaviruses are enveloped viruses, with spherical infectious particles with a diameter of 120 nm; the infectious particle comprises an RNA single-chain positive genome of about 30,000 nucleotides. Specifically, in humans, these viruses were known to cause common colds or seasonal asymptomatic infections, and were considered the causative agents of 15 to 30 percent of common colds. Nevertheless, in the past 20 years, several members of these families have been associated with various acute respiratory syndromes in humans worldwide, now representing an urgent global public health problem.

In 2002, the Severe Acute Respiratory Syndrome Coronavirus (SARS-CoV) generated an outbreak, with more than 8000 confirmed cases, and a fatality rate of ∼9.6% [3]. In 2012, a new coronavirus (MERS-CoV) emerged in the Middle East as the causative agent of a respiratory disease similar to SARS, which has had approximately 2000 confirmed cases, and a fatality rate greater than 40% [4,5]. On December 2019, several pneumonia cases were associated with a new coronavirus strain (SARS-CoV2). The SARS-CoV2 virus is the causal agent of the respiratory disease COVID-19. This virus mainly infects cells of the human respiratory tract. If the infection progresses, it infects pneumocytes, which are cells in the lower airways, causing severe and deadly conditions. The SARS-CoV2 outbreak caused the pandemic that, by the end of March 2021, had infected more than 121 million people in the world, with more than 2.5 million associated deaths [5].

It is urgent to understand these infections’ nature, and the complicated relationships between the host and these pathogens. Among other aspects, it is essential to study the basic biology, epidemiology, and evolution of coronavirus; this will help to cope with the current pandemic, and to be prepared for future events that are a latent danger for humanity.

The discoveries of the scientific research work published in the different biomedical journals represent a valuable source for understanding diseases such as SARS-CoV2, leading to the development of adequate drugs and clinical treatments. However, the accumulation of scientific literature related to the study of different diseases has grown considerably, and coronavirus biomedical literature is no exception [6]. Computational tools represent one way to cope with the enormous costs in time, money, and effort required by the human eye’s exhaustive and systematic review of scientific literature. Through text mining and natural language processing techniques, it is possible to extract knowledge more efficiently [7]. Some successful examples of scientific text mining are represented by the knowledge extraction in the subjects of material science and biomedical science [8,9].

Currently, some applications facilitate researchers in the biomedical area, and other users who are not experts in programming languages, the use of natural language processing techniques and text mining, in tasks that allow the extraction of knowledge from large volumes of information [10]. The knowledge obtained from these applications allows users to make decisions in elaborating new hypotheses or collecting evidence on some phenomena under study [11,12]. Some biomedical applications based on natural language processing and text mining are discussed in the following: ScanGEO, which was developed in Shiny, uses the gene expression omnibus (GEO) databases to find differentially expressed genes based on specific search criteria defined by users [13]. GENETEX is a web application that uses semi-structured input data, such as genomic reports, and, through a series of text mining functions, extracts relevant genomic information in a structured output format [14]. PubTator is a biological data curation database useful in genetic disease analysis, literature-based knowledge discovery, and other text mining functions [15]. This application is used, in turn, by many other applications, such as those related to coronavirus [16,17]. Text mining has also been used in applications related to coronaviruses, for example, Jelodar et al. used these tools to extract discussions about COVID in social networks to apply the topic modeling, later obtaining information on various topics related to the pandemic [18]. Also, natural language processing tools have allowed the development of chatbots that act as virtual doctors, providing health information to patients and users with questions related to diseases [19]. Other important text mining applications related to coronavirus are Litcovid [20] and COVIDScholar [21]. The first one classifies the information in different topics associated with the scientific literature of COVID-19; on the other hand, COVIDScholar, through natural language processing techniques, allows the extraction of relevant information through the synthesis of academic texts associated with COVID-19. In the CO.ME.T.A. web application [22], text mining and natural language processing tools have been applied using a newspapers corpus related to coronavirus to understand the evolution of the epidemic through sentiment analysis, topic detection, and relevant content extraction. A broader compendium of applications focused on the study of coronavirus can be found in the exhaustive reviews of the following works [23,24]. To the best of our knowledge, and according to exhaustive reviews [23,24], most of the applications dedicated on the study of coronavirus are focused on the summary of information, extraction of concepts, and detection of topics. The analysis of a scientific corpus is scarcely addressed to the detection of relevant genes associated with coronaviruses. KnetMiner [25] is among the few applications observed to study the association of genes and COVID-19 through co-occurrence. However, it is not freely available to study the different Betacoronavirus Genus members, such as MERS and SARS-CoV1. Another application that addresses the detection of relevant associations of genes with coronaviruses through co-occurrence networks is related to the work of Oniani et al. [26]. However, the presentation of the results is not friendly for non-expert users, and depends on external databases to identify genes within the scientific literature. Both previous applications lack the latent semantic analysis (LSA) approach, which has been observed to outperform simple co-occurrence approaches [27] in identifying significant gene associations. The LSA approach has been assessed in the relationships of genetic interactions in gold-standard databases that collect manual information from experimental data [27,28]. Given the above, it is essential to develop applications that have the function of finding relevant associations of genes and the different syndromes related to coronaviruses through validated and freely accessible techniques that could help to better understand the COVID (and related diseases) pathology, and help in improving the diagnosis and drug development.

Also, despite a large number of studies on coronaviruses, the mechanisms of pathogenicity in humans are not fully understood, and even though these studies have increased considerably with the last outbreak of SARS-CoV2, the drug design or treatments for the diseases associated with this viral family are poorly developed [29,30]. Due to the needs mentioned above, related to the current pandemic and possible future outbreaks of coronaviruses, we consider it imperative to develop a web application capable of identifying relevant associations of genes with the different syndromes related to coronaviruses. These relevant associations can be used in understanding infection and pathogenicity mechanisms, giving clues about potential diagnostic markers, molecular drug targets, and future treatments.

In the present work, we present a web application employing text mining techniques and natural language processing that allows the extraction and association of potential molecular targets (genes) by analyzing the latent semantic space, and which uses metrics such as cosine similarity. Also, this system lets us view and interact with the most outstanding gene networks associated to coronavirus diseases, and download each of these gene’s information. From an application point of view, identifying relevant genes to coronavirus-associated diseases is of great importance for clinicians and health scientists. Specifically, the present work streamlines the detection of relevant genes associated with coronavirus diseases, since health professionals can extract relevant knowledge without possessing programming skills, reading article by article, or employing large numbers of people, saving time and money. Furthermore, we provided a list and description of genes with outstanding associations to COVID-19, presented in an integrative and summarized way, useful for domain researchers. Another aspect to highlight is that the application shows a more extensive list of genes that can be explored through hypotheses and experiments. Additionally, the web application allows the generation of article filters by genes or keywords, and the detection of diseases and genes associated with each article, according to the PubTator database, a web-based system for assisting bio-curation [15].

## 2. Materials and Methods

By means of the bonafide biomedical literature database PubMed [31], abstracts were extracted using the mesh-controlled vocabulary, using “coronavirus” as a keyword to retrieve papers published from January 2002 to October 2020. From the abstracts obtained, the text was processed to extract relevant terms/genes. The terms were selected using the labels of the most pertinent syndromes associated with coronavirus. Specifically, the chosen coronavirus-related syndromes were the Middle East Respiratory Syndrome (MERS-CoV) and Severe Acute Respiratory Syndrome (SARS-CoV, SARS-CoV2). Additionally, gene occurrence was evaluated according to the uniport gene list. By extracting the previous components, a document-term/gene occurrence matrix was generated using the pubmed.mineR library [32]. This occurrence matrix was used to find associations between document-terms/genes through the corpus associated with the coronavirus genus members through the LSA [33], and on the raw matrix, employing cosine similarity. Previously, the LSA has been used successfully to find both known (explicit) and unknown (implicit) relationships between genes by decomposing the document-term matrix’s singular values extracted from a large corpus of scientific literature [28,34]. The LSA can be used as a useful distance metric, and has been shown to stand out from other approaches, such as co-occurrence models and simple spatial vector models (VMS), when evaluated on gold-standard data sets [27]. Derived from the recognized utility in the prediction of gene–gene and gene–keyword relationships, in the present work, we decided to implement the LSA in a web application allowing us to extract knowledge about the most relevant syndromes associated with coronaviruses. After calculating the latent semantic space with the LSA r library [35,36], the cosine similarity measure was used to find the association of genes, which is considered one of the most widely-used and optimal for this type of analysis [37].

In addition to the association between terms/genes, this work implements filters and data visualization options through an interactive web application implemented in the Shiny Dashboard. Shiny is a package developed by RStudio based on reactive programming that integrates CSS, JavaScript, and HTML code, making it easier for users to interact with data without manipulating the code, ideal for people in the biological areas and biomedical sciences with little expertise in programming [38]. ScanGEO [13], GENETEX [14], shinyCurves [39], COVISA-19 [40], and CO.ME.T.A. [22] are successful examples of web applications developed in Shiny. These display options that allow us to interact with the generation of a subcorpus using keywords, tables, and networks of gene/term associations interactively, and a cloud of words and genes associated with the corpus. Their genes and associated diseases can also be extracted from the selected documents according to the PubTator database, in addition to the downloaded gene information retrieved from the UniProt database [41].

**Figure 1:**
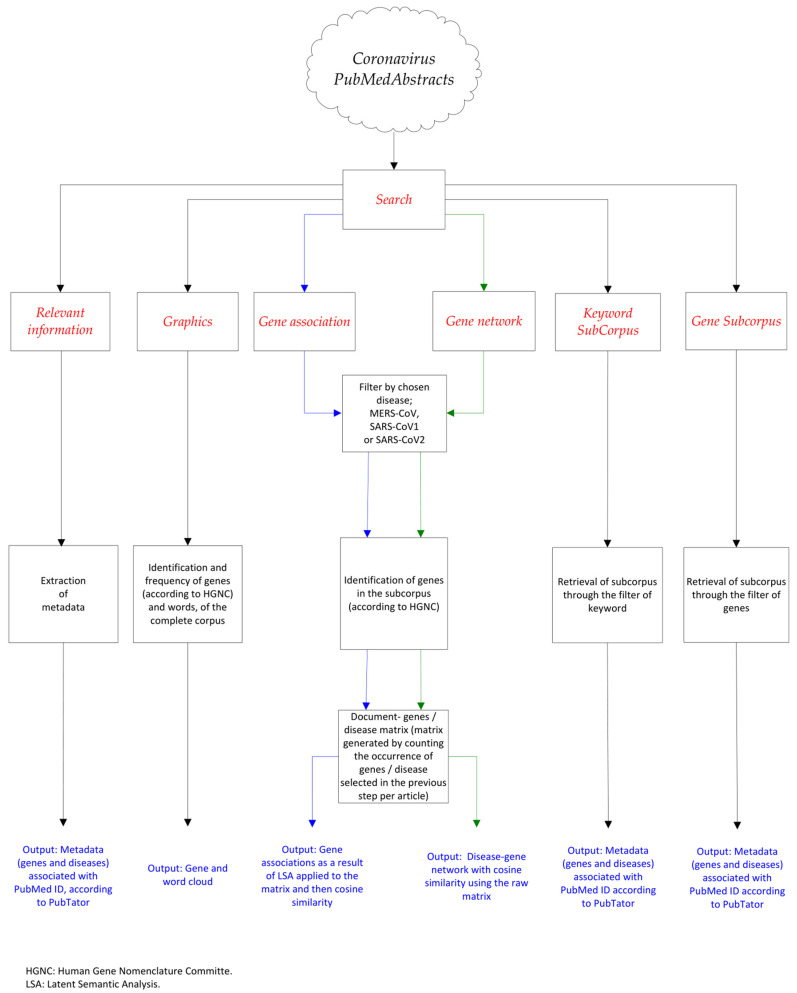
Diagram of the main menu, tools, and technologies used for the interactive web application, named Coronavirus Finder. The blue arrows indicate the process carried out when activating the Gene Association function and the green arrows when the Gene Network function is activated.

In Figure 1, the options in the main menu of the web application are shown in red. The use of the search function is necessary for the proper initialization of the menu options. The flow chart shows all the processes when activating the menu options. For example, in the case of the activation of the gene association menu, the subcorpus associated with the selected disease is filtered, using the *searchabsL* function, and also the associated genes are extracted using the *gene_atomization* function, both functions from the pubmed.mineR library. From the above procedure, a document-terms/genes occurrence matrix is obtained, with which a latent semantic analysis is carried out later with the LSA function of the library of the same name. Subsequently, to the matrix from the LSA, the measure of similarity by cosine between the genes and the chosen disease is calculated. According to cosine similarity, the genes with the highest association suggest being the most related to the disease, and are displayed in a table within the web application. Additional menu options that display metadata associated with articles use the PubTator database, a web-based system that assists bio-curation. The web application is accessible for free at the following address: https://exploration88.shinyapps.io/CoronaFinderA/ (accessed on 17 January 2022).

## 3. Results and Discussion

Combining natural language processing techniques, computational intelligence, and web development tools, it was possible to create an interactive application that allows unraveling relevant and up-to-date information related to the infection caused by coronaviruses that is essential to human health.

**Figure 2:**
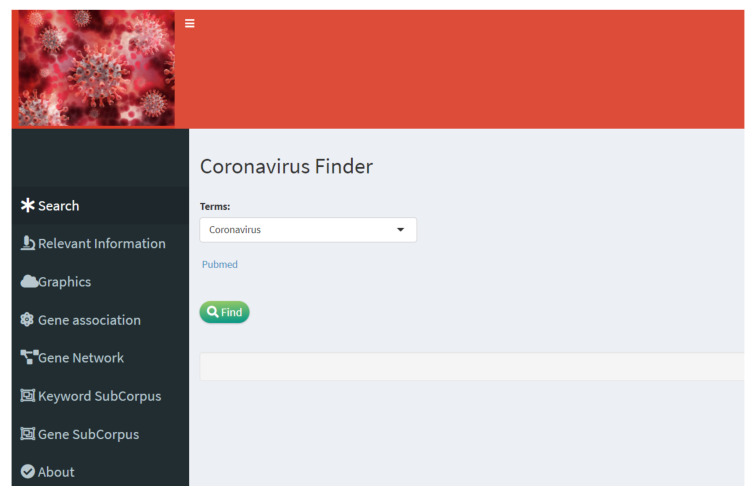
Front-end and menu of the interactive web application, named Coronavirus Finder.

The front end of the web application can be seen in Figure 2, which shows the main menu.

**Figure 3:**
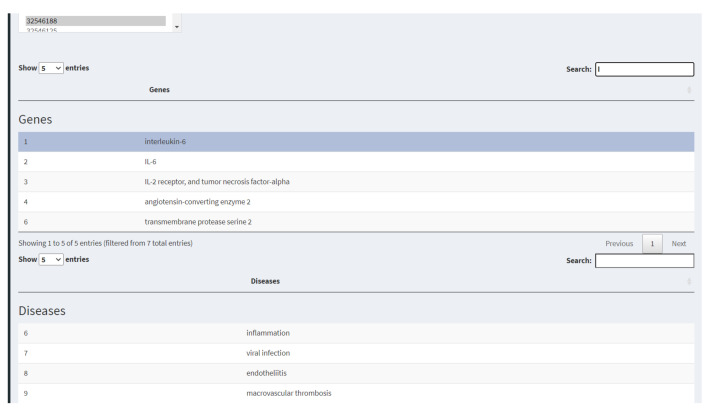
*Relevant information*, to see the metadata of genes associated with an article, selecting its PubMed ID.

On the left side of the application, there is access to the different tools enclosed in the main menu: (1) *Relevant information* makes a description of the scientific articles with associated metadata on genes and diseases for each article according to the PubTator database. (2) *Graphics* generates a word or gene cloud with options for the number of words and geometric figures. (3) *Gene association* finds the association between genes and some of the three selected viruses (SARS-CoV1, SARS-CoV2, and MERS-CoV) through latent semantics analysis. (4) *Gene network* generates the association network based on cosine similarity between all genes and the selected diseases. (5) *Keyword and gene subcorpus* allow filtering by keywords and genes; the resulting subset of articles are extracted from the information of genes and associated diseases according to the PubTator database.

### 3.1. Relevant Information

This tool extracts the gene and disease information of each scientific article, according to its PubMed ID, in the PubTator database. The extraction is useful to promptly enlist the diseases and genes associated with each research article (Figure 3). The menu also displays a text box (*Search* option) to identify specific terms within the results.

### 3.2. Graphics

**Figure 4:**
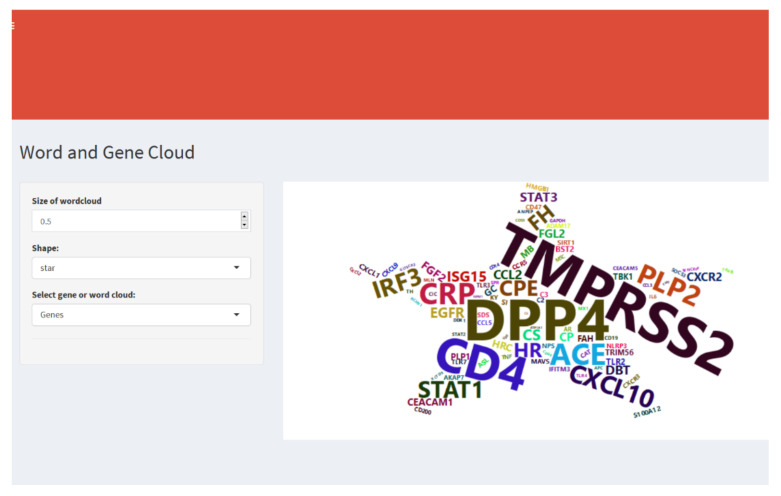
Menu and settings to generate the word cloud in different geometric figures. It is possible to select the option “Word” to generate the cloud of the most frequent words present in the abstracts of coronavirus papers. The “Genes” option shows the cloud of the most frequent genes present in the abstracts. The “Size of wordcloud” option refers to font size: the default is 0.2, and a larger size means bigger words.

In the *Graphics* option (Figure 4), the web application generates a word or gene cloud extracted from scientific articles employing text mining techniques. Also, there are settings such as font size and five geometric shapes for text clouds to display the most abundant words or genes in the body of biomedical literature associated with the coronavirus.

### 3.3. Gene Association

**Figure 5:**
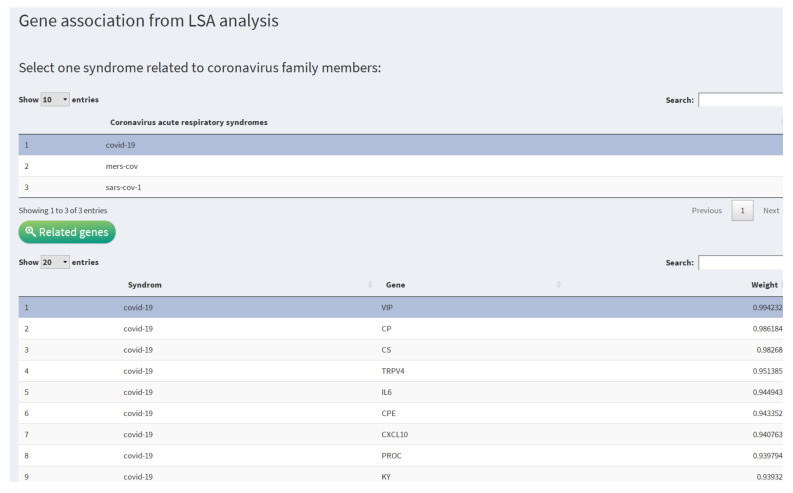
Association of genes with coronavirus diseases according to latent semantics analysis.

The latent semantic analysis of the scientific literature gives us the genes with the most significant association with the three most relevant human diseases caused by coronaviruses. For example, Figure 5 shows the top genes associated with the SARS-CoV2 disease (COVID-19). In this figure, we can observe the following results:

#### 3.3.1. Vasoactive Intestinal Peptide

As the top one of the genes associated with SARS-CoV2, the vasoactive intestinal peptide (VIP), a 28 amino acid peptide that belongs to the class II G-protein ligand-coupled receptors, stands out [42]. Since the 70s, this gene has been shown to protect the lung from other infectious and immune system damages [43]. Interestingly, this gene is also considered a potential repurposed drug target for the critical treatment of COVID-19 in patients with respiratory failure. Currently, there are phase II trials to validate the synthetic gene of VIP (Aviptadil) with the Food and Drug Administration (FDA), conducted by researchers from the University of California, Irvine [43].

#### 3.3.2. Ceruloplasmin

Another outstanding gene associated with COVID-19 is ceruloplasmin (CP), a ferroxidase-type protein that participates in iron metabolism, and its primary function is copper transport. In vitro evidence has suggested that ceruloplasmin helps defend the host by balancing ferritin levels, favoring the anti-inflammatory response. Specifically, it has been seen that it interacts with lactoferrin in the transference of ferric iron, avoiding the formation of toxic hydroxyl radicals [44]. Derived from its function, the modulation of ceruloplasmin activity is desirable when developing new drugs, in conjunction with copper administration, since both have been shown to favor cell antiviral defense [29,45] in candidate treatments against COVID-19. Other authors directly suggest lactoferrin as effective against oxidative stressors such as COVID-19 [46,47].

#### 3.3.3. Transient Receptor Potential Vanilloid

Similarly, one of our genes within the top ten in association with COVID-19 is the transient receptor potential vanilloid 4 (TRPV4) calcium-permeable ion channel. TRPV4 inhibition decreases the pathology in lung edema models, and its overactivation damages the alveoli–capillary barrier [48]. In this sense, TRPV4 is considered a potential approach in treatments against SARS-CoV2 through its protective effects of the alveoli–capillary barrier [48].

#### 3.3.4. Interleukin 6

Interleukin 6 (IL-6) encodes a cytokine associated with inflammation and maturation of B cells. This protein is mainly concentrated in acute and chronic inflammation sites, and is produced mainly by cells of the immune system and almost all stroma cells [49]. Several studies have shown that high levels of IL-6 are associated with SARS-CoV-2 infections and lung lesions in SARS-CoV-2 patients [50,51]. Besides, the monoclonal Tocilizumab antibody against the IL-6 receptor has been used as an option in patients with substantial lung injuries in Italy (TOCIVID-19 study) [52]. This treatment is suggested only when there is clinical and radiological evidence of lesions in the lungs, since in different models, it has been observed that IL-6 is a fundamental cytokine in the early stages for containing the development of different infectious diseases [51].

#### 3.3.5. CXCL10

Like IL-6, another gene appears within the top associations, C-X-C motif chemokine 10 (CXCL10), which encodes a cytokine with a pro-inflammatory response, with a well-established role in the COVID-19-related cytokine storm and severe lung damage [53]. Recent studies have found that IP-10 (interferon gamma induced protein-10), also known as CXCL10, appears to be a critical factor in exacerbating acute respiratory distress syndrome (ARDS) pathology [49]. Due to the above mentioned, it has been proposed that modulators that target CXCL10 may be promising treatments in the acute phase of ARDS to ameliorates acute lung injury in COVID-19 patients [54].

#### 3.3.6. Protein C

This gene encodes a coagulation factor, and plays an important role, regulating anticoagulation, inflammation, cell death, and maintaining the permeability of blood vessels’ walls in humans. Protein C is a vitamin K-dependent glycoprotein that circulates in blood plasma. The active protein C (APC) is generated through the thrombin–thrombomodulin complex; it stands out for its ability to regulate various host defense subsystems, such as those related to inflammation and coagulation. In preclinical studies, APC reduces excessive inflammation and thrombin generation, reducing damage to various organs, including the lungs, and reducing deaths by bacterial pneumonia [55].

#### 3.3.7. SRM

Another relevant gene is SRM, encoding the Spermidine synthase enzyme, which catalyzes spermidine production from putrescine and decarboxylated S-adenosylmethionine (dcSAM). Previous studies show a potential decrease in the expression of Spermidine synthase mediated by the SARS-CoV2 virus, which, in turn, is reflected in a decrease in the spermidine metabolite, resulting in a decrease in the autophagy process [30]. The autophagy process slows the spread of the SARS-CoV2 virus. One way to reverse it is through exogenous supplementation of spermidine, which has been shown to inhibit the spread of SARS-CoV2 by 85% [30]. Additionally, modulators that increase spermidine synthase activity could be developed by further understanding spermidine production, being desirable as a potential treatment against COVID-19.

#### 3.3.8. CYP3A4

The Cytochrome P450 3A4 (CYP3A4) gene is a member of the cytochrome P450 oxidizing enzyme family. This gene is associated with the metabolism of organic molecules such as drugs and xenobiotics. Specifically, drugs associated with COVID-19 treatment, such as atazanavir and lopinavir/ritonavir, inhibit CYP3A4, and others, such as hydroxychloroquine, are metabolized by this cytochrome [56]. Due to their relevance in various drugs’ pharmacokinetics, CYP3A4 inhibitors, such as cobicistat, have been used in combination with other drugs in COVID-19 clinical trials with the intention to avoid their premature degradation, and to favor their action [57].

#### 3.3.9. HMGB1

High mobility group box-1 (HMGB1) is a peptide with cytokine activity. The overexpression of the ACE-2 receptor has been associated with a decrease of HMFB1 expression in mouse models, leading to the hypothesis that the reduction caused by ACE-2 induced by the virus increases the levels of HMGB1, contributing to the cytokine storm in COVID-19 infection [58]. Due to this, various authors have suggested in-depth clinical studies using the HMGB1 peptide as a drug target for the treatment of inflammatory processes associated with COVID-19 [59].

#### 3.3.10. NLRP3

NLRP3 (NOD-, LRR-, and pyrin domain-containing protein 3) is a sensor that detects different endogenous and environmental danger signals, which, when activated, result in the formation of the NLRP3 inflammasome. The NLRP3 inflammasome leads to caspase 1-dependent release of pro-inflammatory cytokines IL-1β and IL-18, favoring the cytokine storm [60]. The overproduction of TNF-α in COVID-19 preferentially activates the NLRP3 inflammasome relative to other immunological pathways. The study relevance and development of drug targets for this pathway have been suggested [60,61]. There are currently different therapeutic molecules under development in different clinical study phases that aim to suppress this pathway.

Interestingly, most of the relevant genes associated with COVID-19 are related to inflammatory processes in response to the virus, specifically to the cytokine storm, which is considered one of the most detrimental processes in patients’ pathology. The information described above strongly demonstrates the efficacy of the Coronavirus Finder web application on the detection of cellular components strongly associated with the process of infection by coronaviruses; more importantly, these data confirm the viability of this web tool as a potential identifier of cellular drug targets, for the understanding or design of treatments against infections associated with coronaviruses.

### 3.4. Gene Network

**Figure 6:**
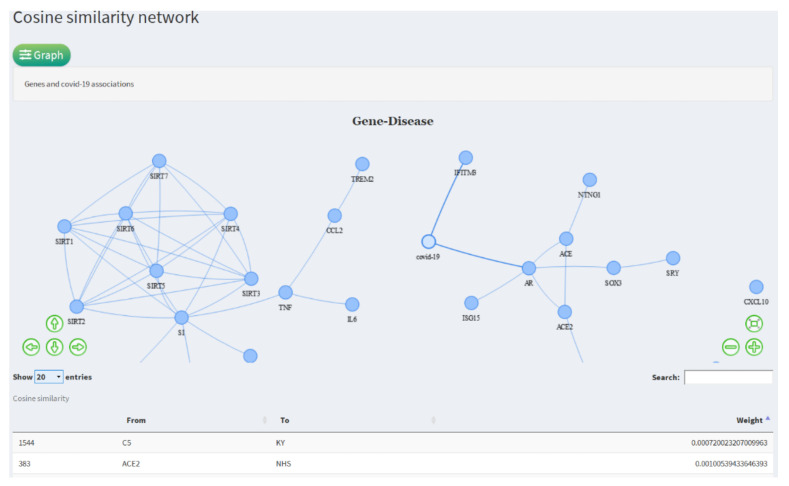
Disease–gene association network obtained by cosine similarity.

As a complement to the LSA–cosine approach, this tool generates a network from the gene/term-document raw occurrence matrix, applying the cosine similarity measure. The network is built considering the pairs with the most outstanding similarity values where the nodes are (genes/term), and the edges between nodes are established if the cut-off value of similarity between pairs of genes and terms is satisfied. In the network obtained, we can observe two principal gene modules (see Figure 6). The module on the left is made up of the SIRT protein family, encoding the functions of seven mono-ADP-ribosyl transferases and NAD + - dependent deacylases.

#### 3.4.1. SIRT Protein Family

Together, this family of proteins is involved in metabolic regulation, inflammatory response, and the first defense line against viral pathogens [62]. The exacerbated inflammatory response in COVID-19 is associated with deficiencies in NAD+. Their levels decrease with age and in conditions associated with oxidative stress, diabetes, and hypertension, the same groups of patients that have high mortality [63]. Because members of the SIRT family are dependent on the availability of NAD+, decreases in this molecule impair its activity, causing a hyper-inflammatory response. To minimize these responses’ impact, some authors have suggested a nutritional supplement with NAD+ precursors and activators of the protein SIRT family [63].

#### 3.4.2. TNF

Higher levels of tumor necrosis factor (TNF), a pro-inflammatory cytokine, have been associated with increased COVID-19 mortality. Observational studies on anti-TNF treatments, used in different previous diseases, have shown favorable results in the development of the pathophysiology associated with COVID-19. Derived from the above, studies have been generated to evaluate the repositioning of anti-TNF therapies in COVID-19 treatments [64].

#### 3.4.3. TREM2

In Figure 6, related to the SIRT protein family module, we found the Triggering Receptor Expressed on Myeloid Cells 2 (TREM2) gene, which encodes a membrane receptor protein that participates in the immune and inflammatory response related to the production of cytokines and the cytokine storm. Due to the above, molecular targets associated with TREM2 have been proposed that affect its activity (specifically in the test phase): inhibitory molecules of galectin-3, and the activator of TREM2 [65].

#### 3.4.4. CCL2

Connected to TREM2, we found the small inducible cytokine A2 (CCL2) in its mature form: it is a 76 amino acid protein involved in immunoregulatory and inflammatory processes. In critically ill COVID-19 patients, the expression of CCL2, in conjunction with its receptor CCR1, has been shown to be significantly increased [66]. Due to their relevance in inflammation processes, some CCL2 inhibitors are under study, and have shown favorable results in vitro [67].

The module on the right of Figure 6 shows genes more closely related to COVID-19, such as its ACE2 receptors that facilitate the virus’s entry.

#### 3.4.5. AR

The AR gene is also closely related to the COVID-19 module, which encodes the androgen receptor protein, and is activated by androgen hormones such as testosterone [68]. The primary function of the androgen receptor is as a DNA-binding transcription factor regulating gene expression. Because AR regulates the expression of the SARS-CoV2 ACE-2 receptor and Transmembrane protease serine 2 (TMPRSS2), both directly involved in the virus infection process, various authors have suggested that it could be involved in the gender difference with respect to the severity of COVID-19, where men have higher mortality than women [69]. Besides, the authors suggest that if androgen sensitivity is confirmed as a predisposition to COVID-19, the use of anti-androgens or androgen modulator drugs as treatments could be used as a potential strategy [67,69].

#### 3.4.6. ISG15

SG15 encodes an interferon-induced ubiquitin-like protein present in the COVID module connected to the AR gene. Expression of ISG15 is induced by type I interferon (IFN-α/β) signaling, and is involved in defense processes in the immune response against viral infections through inflammatory processes [70]. In regard to the papain-like protease PLpro protein, in addition to being essential in SARS-CoV2 replication, it has been observed that its inhibition limits the secretion and extracellular signaling of ISG15. Therefore, therapeutic inhibition of PLpro might be beneficial to COVID-19 patients by decreasing the activity of pro-inflammatory cytokines [71].

#### 3.4.7. IFIT

The IFIT gene, which is also present in the COVID-19 module, encodes the interferon-induced protein with tetratricopeptide repeats known for their broad spectrum of antiviral functions. IFIT has been observed to inhibit cellular entry of SARS-CoV1 and MERS-CoV [72]. Due to the high percentage of identity between the coronaviruses COVID-19 and SARS-CoV1, it has been predicted that IFTM could be a target of studies aimed to develop protecting therapies against the invasion of SARS-CoV2 [73].

#### 3.4.8. SRY and SOX3

Interestingly, the SRY and SOX3 genes, related to the COVID-19 module in the network, are observed. Although the ACE2 receptor is located on the X chromosome, its activity could be decreased by the SRY and SOX3 genes present on the Y chromosome [74]. Therefore, it has been suggested that these genes could be directly or indirectly impacting the balance of ACE1 and ACE2 receptors, which, in turn, influences the response to COVID-19 [75]. It has been observed that men have more significant complications, and are 1.5 to 2 times more likely to die from COVID-19 than women. Also, in dysregulation of the Y chromosome, the SRY gene in older adults increases testosterone levels. Increased testosterone, in conjunction with low estrogen levels, is a disadvantageous factor in various diseases, such as heart diseases [76]. In-depth studies of these genes could reveal important clinical aspects in COVID-19 syndrome.

Finally, we observe that the raw–cosine approach is less permissive than the LSA–cosine approach, since the values of the associations between genes are low. This effect may be due to the lack of dimensionality reduction of the raw–cosine approach, causing a sparser matrix. Although a traditional cosine measure is commonly used to determine the similarity between vectors, it is known that it does not care much about how many features two vectors share. Despite the above, the highest raw–cosine association values show the functional gene relationships previously described, suggesting that it complements the LSA–cosine model.

### 3.5. Gene and Keyword Subcorpus

**Figure 7:**
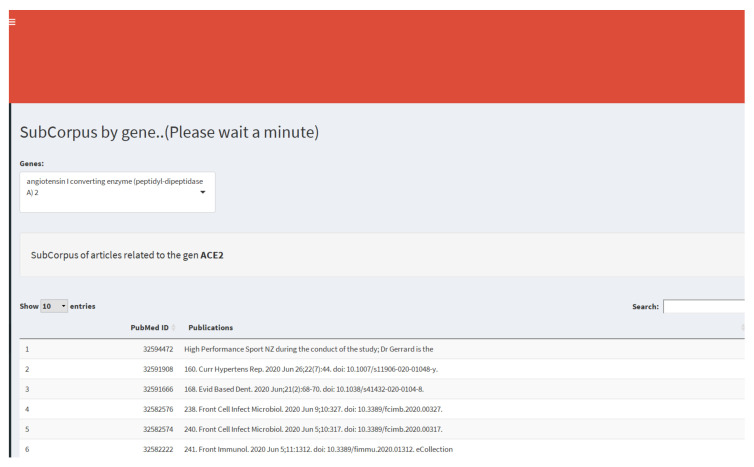
Filters by gene or keywords.

**Figure 8:**
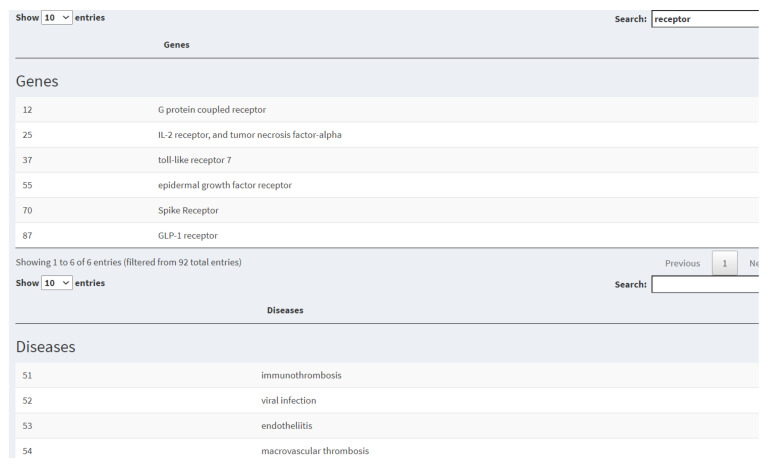
Tables of Genes and Diseases associated with specific gene search according to PubTator database. In the search box of the Genes and Disease tables, it is possible to locate specific terms, as can be seen with the “receptor” search in the first table of the figure.

The gene and keyword subcorpus tools are filters that allow us to generate subsets of documents associated with coronavirus according to the keywords or genes found in the abstracts of papers. The PubMed ID number of articles returned by the gene search are used as a PubTator database input to retrieve the associated diseases and genes. For example, using the filter shown in Figure 7, we can search for articles in our coronavirus database that contain a particular gene, such as ACE2. ACE2 encodes the Angiotensin-converting enzyme 2 protein, and its particular interest lies in that it serves as the entry point into cells for SARS-CoV1 and SARS-CoV2. Additionally, we obtain a data table of genes and diseases associated with the ACE2 gene’s scientific literature according to the PubTator database (see Figure 8).

In summary, we provided a list and description of genes with outstanding associations with COVID-19 presented in an integrative and summarized way useful for domain researchers. Without our tool, this task would take a lot of effort and time for a reader in this domain. Another aspect to highlight is that we describe the relationships with the best score, which could represent the most obvious (explicit) associations, but the application shows a more extensive list of genes that can be explored through hypotheses and experiments. In this regard, the LSA approach has also revealed important implicit gene associations, which are indirect relationships that can be investigated in depth [28]. From all the genes mentioned and identified in the application, many of them are under research processes for drug development; in addition, although many drugs already developed for the disease are directed against proteins from the virus itself [77], these other drugs being investigated can improve the treatment of COVID patients in the near future.

Finally, we want to emphasize the following contributions of this research: first, a web application was developed that comprises scientific literature associated with virus members of the Genus Betacoronavirus, responsible for emerging diseases on human health. Second, the information-compiled web app aims to understand the basics of these coronavirus infections, and the nature of their pathogenesis. Third, our gene association analysis reveals drug targets in understudies, and new candidates suggested in the scientific literature to treat coronavirus, enabling the identification of molecular and cellular components that may function as potential therapeutic targets.

## 4. Future Work

With advances in the field of neural networks, a set of language models has been generated, which represent words as embedding. In these models, natural language words or phrases are represented as vectors of real numbers, and have shown excellent performance in relating words and concepts. Currently, models that combine embedding with attention mechanisms through encoders/decoders, such as Embedding from Language Models (ELMo) [78] and Bidirectional Encoder Representations from Transformers (BERT) [79,80], have had an outstanding performance in different natural language processing tasks. Specific models have also been developed for the biological field, such as Representations from Transformers for Biomedical Text Mining (BioBERT) [81], which has shown significant potential. Even though some models, such as BioBERT, have been evaluated in tasks such as ration extraction, they have not been exhaustively evaluated in manually curated databases [82], such as in the LSA approach. The LSA approach has been evaluated in the relationships of genetic interactions in gold-standard databases that collect manual information from experimental data, such as those from gene expression [27,28]. We are currently planning to evaluate some models generated with embedding, using bonafide databases as benchmarks, to compare them with previous results obtained with the LSA approach. The best performing embedding models in these manually curated gold-standard databases will be added in future deployments of the web application.

## 5. Limitations

Because the corpus used for coronavirus analysis comes from the PubMed database, the bonafide database of the biomedical literature, the web application only considers abstracts in the English language. In addition, abstracts in less-standardized databases were not included for the analysis in the web application, although probably with less relevance to the study of coronavirus. In future works, we consider complementing the abstracts with other databases. Another important problem we face is the variation in the names related to the different syndromes, for example, COVID, SARS-CoV2, COVID-19, SARS-CoV-2, among others. If any of the most popular terms for the disease are left out, the co-occurrence of genes with the disease will be underestimated, which could affect the value of the associations obtained. The previous problem was minimized by using a dictionary of the most popular synonyms for the different syndromes related to coronaviruses according to their appearance in PubTator.

## 6. Conclusions

Due to the prevailing need for a platform that facilitates the massive study of the scientific literature associated with coronaviruses, we have decided to develop a web application that provides this function for free to practitioners and the research community. The present web application reveals relevant information associated with genes and syndromes that facilitate the search for people interested in the coronavirus study, making text mining a powerful tool in the extraction of knowledge related to the understanding of pathogenesis, and the discovery of new treatments. Specifically, our work streamlines the detection of relevant genes (potential drug targets) associated with coronavirus diseases, since health professionals can extract relevant knowledge without possessing programming skills, reading article by article, or employing large numbers of people, saving time and money. The web application will be updated periodically to attach the new information of the articles and their associated discoveries with the coronaviruses’ genus. We are currently developing and testing new semantic models for information representation, which will allow us to increase our capacities to extract knowledge based on scientific articles for future versions of the web application.

## Data Availability

All the information was taken from PubMed, which is a public database.
